# Complete Genome Sequence of Lactobacillus reuteri SKKU-OGDONS-01, Isolated from a Chicken’s Small Intestine

**DOI:** 10.1128/MRA.01251-18

**Published:** 2018-11-21

**Authors:** Dongjun Kim, Mun-ju Cho, Seungchan Cho, Yongjun Lee, Sung June Byun, Sukchan Lee

**Affiliations:** a Department of Genetic Engineering, Sungkyunkwan University, Seobu-ro, Jangan-gu, Suwon-si, Gyeonggi-do, Republic of Korea; b Animal Biotechnology Division, National Institute of Animal Science, Rural Development Administration, Kongjwipatjwi-ro, Iseo-myeon, Wanju_Gun, Jeollabuk-do, Republic of Korea; Loyola University Chicago

## Abstract

Lactobacillus reuteri SKKU-OGDONS-01 is a potentially indigenous probiotic strain isolated from the small intestine of a 27-week-old chicken. The complete genome of L. reuteri SKKU-OGDONS-01 comprises a single circular chromosome. Its length is 2,259,968 bp, with a G+C content of 38.9%.

## Abstract (precis)

Lactobacillus reuteri SKKU-OGDONS-01 is a potentially indigenous probiotic strain isolated from the small intestine of a 27-week-old chicken. The complete genome of L. reuteri SKKU-OGDONS-01 comprises a single circular chromosome.

## ANNOUNCEMENT

Probiotics are live microorganisms, which when administered in an adequate amount can confer benefits to the host (1, 2). In the past, the use of antibiotics (antibiotic growth promoters) in feed additives was prohibited due to concerns about antimicrobial resistance in animal microbiota. As such, there was a need to find alternatives, such as probiotics, that could enhance the immunity of farm animals to fight against various pathogens (3–5). To find candidates for feed additives, indigenous Lactobacillus spp., which are potential probiotics, were isolated from the small intestine of a 27-week-old female chicken. The study protocol and standard operating procedures were reviewed and approved by the Institutional Animal Care and Use Committee of the National Institute of Animal Science in South Korea (approval 2018-273). To isolate intestinal bacteria, the small intestine of a chicken was harvested and the sample homogenized using 1.6-mm stainless steel beads. After spreading the homogenized tissue on de Man-Rogosa-Sharpe (MRS) agar, a number of colonies appeared after 2 days. Of the numerous colonies, only one colony was picked from the MRS agar and incubated in MRS medium at 37°C for 2 days. Cells were cultured and later maintained at −85°C using 80% glycerol. To extract the genomic DNA of Lactobacillus reuteri SKKU-OGDONS-01, the bacterial cell wall was lysed using lysozyme and mutanolysin, and genomic DNA isolation was performed using the G-spin genomic DNA extraction kit (iNtRON, Republic of Korea) (6). In order to perform strain identification, we conducted a sequence comparison using the 16S rRNA sequence. Two primers (7), 9F (5′-GAGTTTGATCCTGGCTCAG-3′) and 1542R (5′-AGAAAGGAGGTGATCCAGCC-3′), were used for amplification of the 16S rRNA gene, followed by sequencing via the Sanger/capillary sequencing method. We found that the 16S rRNA gene sequence of L. reuteri SKKU-OGDONS-01 was 99% similar to those of other L. reuteri strains reported in the NCBI database. Construction of the complete genome sequence of L. reuteri SKKU-OGDONS-01 was based on two sequencing methods, using the PacBio RS II platform (Pacific Biosciences, USA) and the HiSeq 2000 platform (Illumina, USA) at Macrogen (Seoul, Republic of Korea). Of the total 151,868 reads obtained after PacBio sequencing, the average read length was 9,424 bp (451× coverage), and these raw sequence data were assembled using the Hierarchical Genome Assembly Process (HGAP3) protocol in the SMRT Analysis software version 2.3.0 (8). Additionally, Illumina sequencing was conducted using the HiSeq 2000 platform. The paired-end reads from a 325-bp insert library were sequenced for L. reuteri SKKU-OGDONS-01. A total of 57,137,834 reads, with an average read length of 100 bp, were generated from Illumina sequencing. After error correction using Pilon (version 1.21) (9) with Illumina short reads, a single contig was generated, at 2,259,968 bp in length, with a G+C content of 38.9%. Using comparative genome analysis, the whole genome of L. reuteri SKKU-OGDONS-01 was about 97% similar to those of other L. reuteri strains, including L. reuteri strain I49 (GenBank accession number NZ_CP015408). Genome annotation was carried out using the NCBI Prokaryote Genome Annotation Pipeline (PGAP) version 4.6 (10). Based on the PGAP result, the chromosome included 2,165 genes and 1,941 coding sequences (CDS). The whole genome of L. reuteri SKKU-OGDONS-01 consists of 70 tRNA genes and 18 rRNA genes.

### Data availability.

The chromosomal sequence of L. reuteri SKKU-OGDONS-01 has been deposited at GenBank under the accession number CP029615. The sequencing reads (under Sequence Read Archive number SRP162209) can be accessed through BioProject number PRJNA473291 and BioSample number SAMN09270376.
